# Hypogammaglobulinemia in Children with Atopic Dermatitis

**DOI:** 10.3390/children13050696

**Published:** 2026-05-19

**Authors:** Zuhal Karali, Yasin Karali, Zeynep Senocak, Sara Sebnem Kilic

**Affiliations:** 1 Department of Pediatric Immunology and Allergy, University of Health Sciences, Bursa City Hospital, 16110 Bursa, Turkey; zuhal.karali@saglik.gov.tr (Z.K.); zeynep.senocak@saglik.gov.tr (Z.S.); 2 Division of Pediatric Immunology, Faculty of Medicine, Bursa Uludag University, 16110 Bursa, Turkey; yasinkarali@uludag.edu.tr

**Keywords:** children, atopic dermatitis, hypogammaglobulinemia

## Abstract

Background and aims: Atopic dermatitis (AD) is the most common chronic inflammatory skin disease among children. Our study aimed to evaluate the prevalence of hypogammaglobulinemia among pediatric patients with AD and to characterize the clinical and laboratory findings of patients diagnosed with AD and hypogammaglobulinemia. Method: The electronic files of 1850 patients aged 0–18 years diagnosed with AD between 2020 and 2022 in the Pediatric Immunology and Allergy Clinic of Bursa Medical Faculty City Hospital were retrospectively analyzed. During this period, all patients newly diagnosed with atopic dermatitis at our clinic were systematically screened for their serum immunoglobulin (Ig) levels (IgG, IgA, and IgM) at the time of initial presentation. We included 200 AD patients with hypogammaglobulinemia. Disease severity was classified using the Scoring Atopic Dermatitis (SCORAD) index. Multivariate logistic and linear regression analyses were performed to identify independent determinants of disease severity, considering age, sex, eosinophil counts, total IgE, food allergies, and baseline immunoglobulin levels. Results: The prevalence of hypogammaglobulinemia among the 1850 screened children with AD was 10.8% (200/1850). Of the 200 patients included in this study, 128 (64%) were male, and 72 (36%) were female. The median age at first clinic presentation was 8 months (interquartile range (IQR) 25–75%: 5–16). According to the Scoring Atopic Dermatitis (SCORAD) index, AD severity was mild in 150 (75%) patients and moderate-to-severe in 50 (25%). Food allergy sensitization was present in 72 (36%) patients. Patients with moderate-to-severe AD had significantly lower IgG (300 vs. 374 mg/dL; *p* < 0.001; r = −0.346), IgA (10 vs. 14 mg/dL; *p* = 0.004), and IgM (38 vs. 51 mg/dL; *p* = 0.001) levels when compared with those with mild disease. Multivariate logistic regression confirmed that lower IgG was the only immunoglobulin independently associated with moderate-to-severe AD (OR = 1.97 per 100 mg/dL decrease; 95% CI: 1.15–3.39; *p* = 0.013), while food allergy was the strongest independent predictor of the SCORAD index (β = +11.97; *p* < 0.001). None of the patients received intravenous immunoglobulin (IVIG) treatment. Of the 142 patients who underwent serial serum immunoglobulin measurements, 56 (39%) achieved age-appropriate normal IgG levels, while hypogammaglobulinemia persisted in 86 (61%). Conclusions: We found a higher frequency of hypogammaglobulinemia in patients with AD in our study, as compared with previously reported rates of THI in children from the general pediatric population. Although our study showed an increase in IgG levels during the follow-up period in many patients, it emphasizes the need for long-term immunological monitoring, especially in patients with moderate-to-severe AD.

## 1. Introduction

Atopic dermatitis (AD) is the most common chronic, relapsing, non-infectious inflammatory skin disease in children. It affects up to 20% of the pediatric population, with 90% of children diagnosed before the age of 5 years [1]. Several factors contribute to the pathogenesis of AD, including genetic disorders, epidermal barrier dysfunction, increased transepidermal water loss, immune dysregulation, and disruption of the skin microbiome [2]. Atopic diseases can also occur in the context of inborn errors of immunity (IEIs), which result from mutations in genes that regulate host defense and immune function. The triad of eosinophilia, AD, and increased serum immunoglobulin E (IgE) is seen in common allergic diseases. However, it may also be a clinical presentation of an IEI [3,4].

Hypogammaglobulinemia is defined according to the ESID/PAGID diagnostic criteria as a serum IgG level at least two standard deviations (SDs) below the age-adjusted mean reference value [5]. Serum IgG levels in infants are lowest at three to six months of age due to the catabolism of passive maternal IgG antibodies. Subsequently, Ig levels increase as the infant initiates endogenous IgG production. Transient hypogammaglobulinemia of infancy (THI) affects infants between 5 and 24 months of age and is characterized by transiently low levels (two standard deviations below normal) of serum immunoglobulins. Several studies have reported an increased frequency of allergic diseases, including atopic dermatitis, with THI [5,6,7]. In a previous study conducted in Turkey, the frequency of atopic dermatitis in children with THI was reported to be approximately 5% [6].

Atopic dermatitis may occur in certain immunodeficiencies, presenting as an “atopic phenotype” characterized by peripheral eosinophilia and elevated total IgE levels. This phenotype can be observed in conditions such as Netherton syndrome, IPEX syndrome, hyper-IgE syndrome, Omenn syndrome, Wiskott-Aldrich syndrome, and STAT6 gain-of-function mutation [8,9]. The presence of serum total IgE at >2000 kU/L, severe eosinophilia (>1500/μL), lymphocytopenia, neutropenia, thrombocytopenia, anemia, and clinical findings suggestive of immunodeficiency, especially in the first 3 months of life, has been regarded as a red flag [10].

This study aimed to assess the prevalence of hypogammaglobulinemia in a large pediatric AD cohort and to characterize the clinical and laboratory features of patients with coexisting AD and hypogammaglobulinemia, given its potential link to underlying congenital immunodeficiency.

## 2. Method

The electronic medical records of patients aged 0–18 years diagnosed with AD between 2020 and 2022 at the Pediatric Immunology and Allergy Clinic of Bursa Medical Faculty City Hospital were reviewed. This was a retrospective cohort study with a cross-sectional baseline analysis and a longitudinal follow-up subgroup. Atopic dermatitis was diagnosed according to the Hanifin and Rajka criteria [11]. Patients with metabolic disorders, previously diagnosed immunodeficiency, nephrotic syndrome, or chronic conditions that may lead to immunoglobulin loss and secondary hypogammaglobulinemia (such as chronic diarrhea, liver disease, or epilepsy requiring antiepileptic therapy) were excluded. In addition, patients receiving systemic immunosuppressive therapy at the time of immunoglobulin measurement (e.g., systemic corticosteroids, cyclosporine, methotrexate, azathioprine, or biological agents) were excluded to avoid confounding by immunosuppression-associated secondary hypogammaglobulinemia. During this period, all patients newly diagnosed with atopic dermatitis at our clinic were systematically screened for serum immunoglobulin levels (IgG, IgA, and IgM) at the time of initial presentation.

After the exclusion criteria were applied, hypogammaglobulinemia was identified in 200 patients (10.8%) within our cohort of 1850 children with atopic dermatitis who were systematically screened for serum immunoglobulin levels; these patients constituted the final study population. Patients with concomitant reductions in serum IgA and/or IgM levels below −2 SDs of the age-specific reference values were not excluded from the cohort. Serum immunoglobulin levels were interpreted according to age-specific reference ranges established for healthy Turkish children, and values below −2 SDs from the age-adjusted mean were considered low [12].

The primary cross-sectional outcomes were (i) the prevalence of hypogammaglobulinemia among children with AD; (ii) the distribution of immunoglobulin levels by age and disease severity; and (iii) the association of hypogammaglobulinemia with food sensitization, eosinophilia, total IgE, and clinical comorbidities. The longitudinal outcomes, evaluated in the subgroup of 142 patients who underwent sequential immunoglobulin measurements every 3 months, were the change in immunoglobulin levels and the proportion of patients reaching age-appropriate normal IgG values during follow-up.

At each follow-up visit, infection history was assessed through a systematic review of medical records. Secondary bacterial skin infections (infected dermatitis) requiring systemic antibiotic treatment or hospitalization, upper and lower respiratory tract infections requiring antibiotic treatment, and other infections requiring hospitalization were recorded. Information on viral upper respiratory tract infections managed in outpatient primary care settings without hospitalization was not systematically available and was therefore not included in the analysis. Regarding infections, only one patient developed infected dermatitis requiring hospitalization and intravenous antibiotic treatment during the follow-up period.

The severity of AD was assessed using the SCORAD index at the time of first presentation. Patients with a SCORAD index below 25 were classified as having mild AD, those with a score from 25 to 50 had moderate AD, and those with a score above 50 were classified as having severe AD [13,14]. For analysis, the patients were grouped into mild versus moderate-to-severe AD. Atopic dermatitis is a disease characterized by a relapsing course with recurrent exacerbations. In this study, immunoglobulin levels and the SCORAD index were assessed at the time of the initial presentation, reflecting the disease activity at first admission.

Clinical data (demographic information, allergen sensitization findings via skin prick test, history of recurrent or severe infections, other accompanying allergic diseases) and laboratory findings (hemogram, serum total protein and albumin levels, and immunologic tests) were recorded. Serum IgG, IgA, and IgM levels were measured by immunoturbidimetric assay on a cobas 8000 modular analyzer (Roche Diagnostics, Mannheim, Germany), and lymphocyte subset levels (CD3+ T cells, CD4+ T cells, CD8+ T cells, CD19+ B cells, and CD3-CD16+CD56+ NK (Natural Killer) cells) were measured by means of flow cytometry. The following data were collected from electronic files and evaluated retrospectively: allergen-specific IgE, 25-hydroxyvitamin D, vitamin B12, tetanus, and hepatitis B vaccine-specific antibody responses. Specific IgE measurements were performed using the IMMULITE^®^ immunoassay method (Siemens, Washington, DC, USA), and a cut-off value of 0.35 kU/L was used to define allergic sensitization. Allergy testing was performed for cow’s milk, egg yolk, egg white, wheat, peanut, beef, soybean, and house dust mites. A cutoff value of 0.35 kU/L was used to confirm allergic sensitization. All patients underwent a skin prick test. Skin prick testing was performed in the pediatric allergy clinic test room using standardized allergen extracts (Diater Laboratorios, Madrid, Spain). Patients were considered allergen-sensitized if the skin prick test or specific IgE was positive. Elimination diets were implemented for foods with positive test results. In patients who showed clinical improvement, an oral food challenge was performed after 4 weeks to confirm the diagnosis of food allergy.

## 3. Statistics

Descriptive and comparative statistical analyses were performed using IBM SPSS version 28.0 Statistics (IBM Corp., Armonk, NY, USA). In contrast, multivariable logistic regression analyses and data visualization were conducted in Python 3.11 using the statsmodels package. The type I error level was set at α = 0.05, and all tests were two-tailed. Descriptive statistics are expressed as frequencies and percentages for categorical variables and as medians with interquartile ranges (IQRs; 25th–75th percentiles) for non-normally distributed continuous variables. Distribution normality was assessed using the Shapiro–Wilk test. The Mann–Whitney U test was used for comparisons between two independent groups, the Kruskal–Wallis test for comparisons across multiple groups, the Wilcoxon signed-rank test for paired samples, and the chi-square or Fisher’s exact test for categorical variables. Correlations between continuous variables were assessed using the Spearman rank correlation coefficient (r_s_). Effect sizes for Mann–Whitney U comparisons are reported as rank-biserial correlation coefficients (r) and interpreted as small (|r| = 0.1), medium (|r| = 0.3), or large (|r| = 0.5). Multivariable logistic regression was performed to identify independent predictors of moderate-to-severe AD, and multiple linear regression was used to analyze the continuous SCORAD index. The model performance for logistic regression was assessed using the Hosmer–Lemeshow goodness-of-fit test and the area under the receiver operating characteristic (ROC) curve. In contrast, linear regression models were evaluated using the F-statistic and adjusted R^2^ values. Multicollinearity was assessed using variance inflation factors (VIF < 3.0). The results are presented as odds ratios (ORs) or unstandardized regression coefficients (β) with 95% confidence intervals (CIs). All *p*-values are reported to three decimal places; *p* < 0.05 was considered to indicate statistical significance.

## 4. Results

Of the 1850 children with AD who were systematically screened for serum immunoglobulins, 200 (10.8%) met the criteria for hypogammaglobulinemia and were included in this study. Of these 200 patients, 128 (64%) were male and 72 (36%) were female. The median age at first clinic presentation was 8 months (interquartile range (IQR) 25–75%: 5–16). Most patients were infants or young children: 56 (28%) were aged 3–6 months, 81 (40.5%) were 7–12 months, 55 (27.5%) were 13–48 months, and only eight (4%) were older than 49 months. According to the SCORAD index, AD severity was mild in 150 (75%) patients and moderate-to-severe in 50 (25%). Food allergy was present in 72 (36%) patients. The most frequently detected food allergens, in order of frequency, were milk (*n* = 52, 26%), egg yolk (*n* = 48, 24%), egg white (*n* = 43, 21.5%), beef (*n* = 29, 14.5%), peanut (*n* = 23, 11.5%), wheat (*n* = 20, 10%), soy (*n* = 18, 9%), and hazelnut (*n* = 16, 8%). Ten (5%) patients tested positive for house dust mite sensitization. The total IgE level was a median of 23.4 IU/mL (IQR 25–75%: 7.9–67.4). The median immunoglobulin levels were IgG 351 mg/dL (IQR 25–75%: 300–456), IgA 13 mg/dL (IQR 25–75%: 10–20), and IgM 45 mg/dL (IQR 25–75%: 22.8–65.5). The eosinophil count (cells/μL) was 320 (IQR 25–75%: 200–500). Table 1 presents the distribution of immunoglobulin values among patients by age group.

The lymphocyte subgroups were within the normal age range in 185 (92.5%) of the patients. None of the patients received intravenous immunoglobulin (IVIG) treatment. Regarding infections, only one patient experienced an episode of infected dermatitis requiring hospitalization and intravenous antibiotic therapy during follow-up. In most cases, exacerbations of atopic dermatitis responded to topical antibiotic treatment. Among the comorbid allergic diseases, asthma was present in three patients (1.5%), allergic rhinitis in 15 patients (7.5%), proctocolitis in 19 patients (9.5%), and anaphylaxis in three patients (1.5%). The low prevalence of asthma (1.5%) is largely explained by the very young age of the cohort (68.5% under 12 months), since asthma is typically diagnosed at older ages.

**Figure 1:**
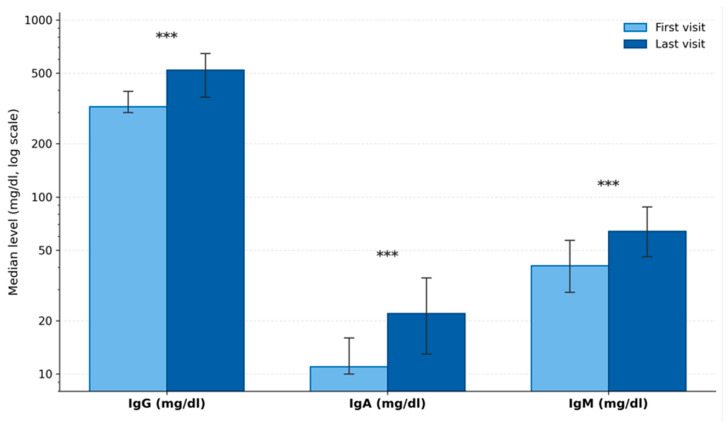
Changes in serum immunoglobulin levels during follow-up in children with atopic dermatitis and hypogammaglobulinemia. Comparison of serum immunoglobulin levels between the first and last clinic visits in 142 patients with serial measurements during a median follow-up period of 18 months (range: 3–25 months). Light blue bars represent baseline (first visit) values and dark blue bars represent last visit values. Bar heights indicate median values; error bars represent interquartile ranges (IQR, 25th–75th percentile). The y-axis is displayed on a logarithmic scale to accommodate the wide range of immunoglobulin concentrations across isotypes. Significant increases were observed in all three immunoglobulin isotypes: IgG (324 to 521 mg/dL; *p* < 0.001), IgA (11 to 22 mg/dL; *p* < 0.001), and IgM (41 to 64 mg/dL; *p* < 0.001; Wilcoxon signed-rank test). *** *p* < 0.001.

Among the 200 patients, 142 underwent sequential serum immunoglobulin measurements at approximately 3-month intervals during follow-up. Among these 142 patients, IgG titers increased from baseline in 125 patients and remained unchanged in 17. However, only 56 of the 142 patients (39%) reached age-appropriate normal IgG levels during follow-up, and hypogammaglobulinemia persisted in the remaining 86 (61%) over a median follow-up period of 18 months (range 3–25 months). There was a statistically significant increase observed in the median immunoglobulin G, A, and M values when the initial immunoglobulin values were compared with the immunoglobulin values at the last visit (*p* < 0.001) (Table 2) (Figure 1). There was no difference in IgG, IgA, or IgM levels among patients with and without food allergies. However, the total IgE level was significantly higher in the group with food allergies. Patients sensitive to two or more allergens had significantly higher total IgE levels compared to those sensitive to a single allergen; however, no difference was observed in their IgA, IgG, or IgM levels (Table 2).

**Figure 2:**
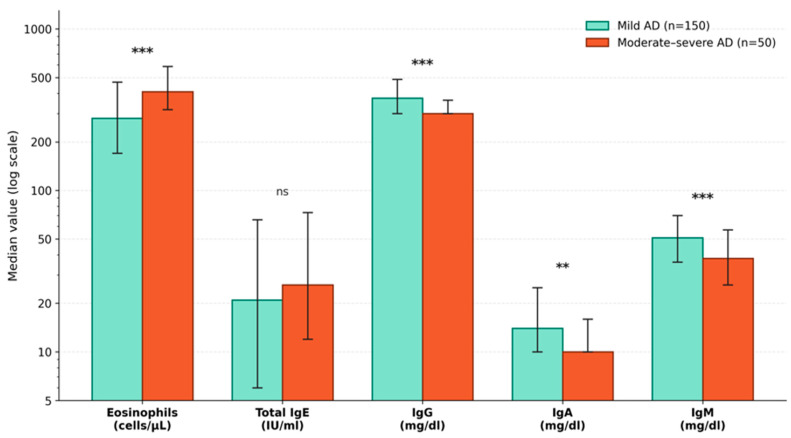
Comparison of laboratory parameters between mild and moderate-to-severe atopic dermatitis groups classified by SCORAD index. Comparison of eosinophil count, total IgE, IgG, IgA, and IgM levels between mild (SCORAD < 25; n = 150) and moderate-to-severe (SCORAD ≥ 25; n = 50) atopic dermatitis groups. Green bars represent mild AD and orange bars represent moderate-to-severe AD. Bar heights indicate median values; error bars represent interquartile ranges (IQR, 25th–75th percentile). The y-axis is displayed on a logarithmic scale. Eosinophil count was significantly higher in the moderate-to-severe group (*p* < 0.001; r = 0.332), while IgG (*p* < 0.001; r = −0.346), IgA (*p* = 0.006; r = −0.268), and IgM (*p* = 0.001; r = −0.319) levels were significantly lower, all with medium effect sizes. Total IgE did not differ significantly between groups (*p* = 0.462). Mann–Whitney U test. *** *p* < 0.001; ** *p* < 0.01; ns, not significant.

The SCORAD index was not significantly different between male and female patients (*p* = 0.29), but it was significantly higher in children with food allergies compared to those without (*p* < 0.001). The mean vitamin B12 and 25-hydroxyvitamin D levels were normal in all groups. The moderate-to-severe group had a significantly higher median eosinophil count than the mild group (*p* < 0.001). The median IgG, IgA, and IgM levels of patients with moderate-to-severe AD were significantly lower than those of patients with mild AD (Table 3) (Figure 2). The differences in IgG (r = −0.346), IgM (r = −0.319), and eosinophil count (r = 0.332) between the mild and moderate-to-severe AD groups corresponded to medium effect sizes, indicating that these associations were not only statistically significant but also clinically meaningful. In contrast, total IgE showed a negligible effect size (r = 0.070), confirming the absence of a meaningful difference between the severity groups (Table 3).

Multivariate logistic regression analysis was performed to identify independent predictors of moderate-to-severe AD, adjusting for age, sex, eosinophil counts, total IgE, food allergy status, and baseline immunoglobulin levels. Among all the covariates, only the IgG level was independently associated with moderate-to-severe disease (OR = 0.993 per 1 mg/dL increase; 95% CI: 0.988–0.999; *p* = 0.013). When expressed per 100 mg/dL decrease in IgG, the odds of moderate-to-severe AD nearly doubled (OR = 1.97; 95% CI: 1.15–3.39) (Table 4). In multiple linear regression with the SCORAD index as the continuous outcome variable (Table 5), food allergy was the strongest independent predictor (B = 11.97; 95% CI: 6.41–17.54; *p* < 0.001), while IgG showed a borderline negative association with the SCORAD index (B = −0.029; *p* = 0.055).

To identify individual-level patterns and extreme-value behaviors that may be masked by group-level summary statistics, scatterplots were constructed to visualize the relationships between the SCORAD index and baseline serum IgG, IgA, and IgM levels, stratified by disease severity.

**Figure 3:**
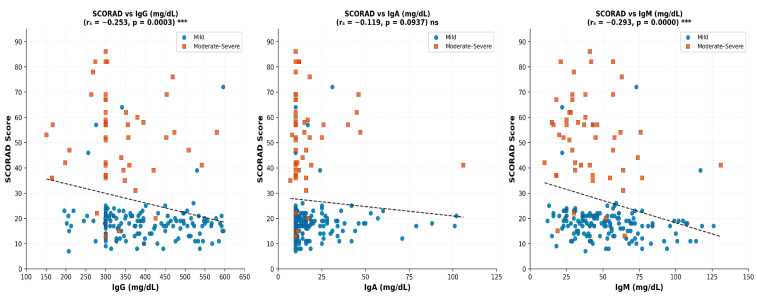
Scatterplots of SCORAD score versus serum immunoglobulin levels stratified by disease severity. Scatterplots depicting the relationships between SCORAD index values and baseline serum IgG (A), IgA (B), and IgM (C) levels. Each point represents an individual patient; blue circles indicate mild AD and orange squares indicate moderate-to-severe AD. Dashed lines represent linear regression trend lines. Spearman correlation coefficients (r_s_) and corresponding *p*-values are displayed. Significant inverse correlations were observed for IgG (r_s_ = −0.253, *p* < 0.001) and IgM (r_s_ = −0.293, *p* < 0.001). Although the Spearman correlation for IgA did not reach statistical significance (r_s_ = −0.119, *p* = 0.094), a group-level comparison revealed significantly lower IgA levels in the moderate-to-severe group compared with the mild group (median 10 vs. 14 mg/dL, *p* = 0.004). This finding suggests that the reduction in IgA does not deepen progressively with increasing SCORAD index value but, rather, manifests as a discrete difference between severity categories. *** *p* < 0.001; ns, not significant (Spearman correlation test).

The scatterplot findings are consistent with and reinforce the results of both the univariate and multivariate analyses. The visual clustering of moderate-to-severe patients in the low-immunoglobulin range corroborates the independent association between lower IgG and disease severity identified through logistic regression (OR = 1.97 per 100 mg/dL decrease) (Figure 3).

Among the 200 patients with low IgG levels, decreased IgA levels were detected in 81 patients (40.5%), decreased IgM levels in 48 patients (24%), and reductions in all immunoglobulin isotypes below −2 standard deviations (SDs) of age-adjusted reference values in 10 patients (5%). Overall, 61 patients (30.5%) had isolated IgG deficiency, whereas 139 patients (69.5%) exhibited concomitant low IgA and/or IgM levels. The characteristics of both groups are compared in Table 6. Baseline IgG levels were significantly lower in the concomitant deficiency group than in the isolated IgG deficiency group (median 336 vs. 399 mg/dL, *p* = 0.014), consistent with the broader immunoglobulin impairment observed in these patients. However, disease severity, food allergy prevalence, SCORAD index values, and total IgE levels did not differ significantly between the groups.

Importantly, normalization of IgG levels during follow-up was significantly more frequent in patients with isolated IgG deficiency than in those with concomitant low IgA and/or IgM levels (53.7% vs. 25.87%, *p* = 0.001).

To further evaluate the impact of the hypogammaglobulinemia subtype on persistent hypogammaglobulinemia, logistic regression analysis was performed. Concomitant hypogammaglobulinemia, defined as low IgG accompanied by low IgA and/or IgM levels, emerged as a significant independent predictor of persistent hypogammaglobulinemia during follow-up (OR = 3.37, 95% CI: 1.21–9.40, *p* = 0.020). These findings support the clinical relevance of subclassifying patients according to accompanying immunoglobulin deficiencies. Notably, although baseline IgG levels were lower in the concomitant deficiency group (median 309 vs. 395 mg/dL, *p* = 0.031), the persistence of hypogammaglobulinemia was more strongly associated with the presence of multiple affected immunoglobulin isotypes than with the absolute IgG level itself (IgG decrease per 100 mg/dL: OR = 1.30, *p* = 0.278).

The most common comorbid conditions were allergic rhinitis (7.5%) and proctocolitis (9.5%). No increase in the frequency of proctocolitis or allergic rhinitis was observed in patients with AD and low immunoglobulin levels. When patients with low levels of IgG, IgA, or IgM were evaluated separately, no difference in allergic comorbidities was observed.

## 5. Discussion

This study examined the prevalence and clinical characteristics of hypogammaglobulinemia in children diagnosed with AD and investigated its relationship with disease severity, food allergies, and comorbidities. Two hypotheses regarding the relationship between AD and immune system defects have been proposed. The first hypothesis is that a defect in the immune system occurs first, followed by the development of an allergen-induced epidermal barrier disorder. The second hypothesis is that a skin barrier disruption occurs first, followed by immune dysregulation [15].

One of the immune system defects associated with AD is THI. It has been reported that AD symptoms may improve after serum IgG levels return to normal [16]. Infants with THI usually present with atopic manifestations such as food allergies, high IgE levels, and moderate-to-severe AD. Although low IgM levels are rare, these patients typically show isolated decreases in serum IgG levels [17]. In our study, the prevalence of hypogammaglobulinemia in children with AD was 10.8%. In contrast, Çeliksoy et al. reported a higher incidence of hypogammaglobulinemia (28/160; 17.5%) in their cohort of pediatric AD patients; however, they did not identify any significant association between disease severity and hypogammaglobulinemia [18]. Notably, our cohort was approximately 11.5 times larger than that in the aforementioned study, thereby providing substantially greater statistical power to detect differences in immunoglobulin levels across severity groups. Importantly, the observation of significantly lower immunoglobulin levels in the moderate AD group in our study suggests that this association may have been underrecognized in smaller cohorts. These findings underscore the value of larger sample sizes in elucidating clinically relevant immunological patterns and lend meaningful support to a potential link between AD severity and humoral immune response.

In our study, patients with moderate-to-severe AD had significantly lower median IgG, IgA, and IgM levels than did those with mild disease. Several non-mutually exclusive mechanisms may underlie this observation. First, severe AD is characterized by extensive disruption of the epidermal barrier and chronic cutaneous inflammation, which may lead to subclinical transcutaneous protein loss, potentially contributing to lower circulating immunoglobulin levels [19]. Second, the marked T-helper-2 (Th2)-skewed inflammatory milieu in severe AD may impair B-cell class switching toward IgG and favor IgE production, a pattern compatible with the elevated IgE and eosinophilia observed in our moderate-to-severe group [20]. Third, severe early-onset atopic dermatitis may be an early phenotypic manifestation of an underlying congenital immune disorder (such as STAT6 GOF, DOCK8 deficiency, Wiskott–Aldrich syndrome, or hyper-IgE syndrome) [21]. From a clinical perspective, children with severe early-onset atopic dermatitis and persistently low immunoglobulin levels require closer immunological monitoring.

Severity classification (mild vs. moderate-to-severe) showed lower IgG, IgA, and IgM levels in the more severe group. In the logistic regression analysis performed in this study, after adjustments for possible confounding factors such as age, gender, eosinophilia, total IgE, and food allergy, IgG was the only immunoglobulin independently associated with AD severity. A complementary finding from the linear regression analysis revealed that food allergy was the dominant independent predictor of the SCORAD index. These two regression models evaluated different but complementary aspects of disease severity. In the logistic regression analysis, low IgG levels emerged as a categorical risk indicator for moderate-to-severe AD; in the linear regression analysis, food allergy was identified as a consistent severity predictor associated with increased SCORAD index.

The unexpectedly low median total IgE (23.4 IU/mL) and the low prevalence of asthma (1.5%) in our cohort, despite AD being classically associated with elevated IgE and atopic comorbidities, can be explained by the very young age structure of our population: 68.5% of patients were under 12 months, and 96% were under 48 months. Total IgE levels physiologically increase with age and with progression along the atopic march [22]. In a study of 52 patients, Berce et al. found that those sensitive to food and/or aeroallergens, as well as those with low serum IgM levels, had more severe AD scores [16]. Consistent with that report, in our cohort, the median SCORAD index value was higher in patients with food sensitization (23, IQR 20–57) than in those without (18.5, IQR 16–22).

Sensitization to foods (e.g., cow’s milk, egg whites, nuts) is common in infants and young children, while sensitization to airborne allergens (e.g., house dust mites, pets, pollen) becomes more common in older children [23]. In our study, 96% of our patients were younger than 48 months, with 68.5% being younger than 12 months. The prevalence of food allergy was 36%, and that of house dust mite sensitization was 5%. The association between food allergies and hypogammaglobulinemia was stronger than that observed with inhalant allergies. Children with AD who are sensitized (polysensitized) to more than five foods and/or aeroallergens are more likely to experience a more severe form of the disease. More than two food allergies were detected in 82 of our patients (41%). While the polysensitization rate was 22.4% in previous studies, our study found a higher rate since we considered at least two food sensitivities as multiple sensitivities [23].

In patients with sensitization to at least two allergens, the SCORAD index was 22 (IQR 25–75%: 18–51), whereas it was 19 (IQR 25–75%: 15–23) in monosensitized patients, and this difference was statistically significant. When monosensitized patients were compared with those sensitized to at least two allergens, IgE levels were significantly higher in the latter group. At the same time, no difference was found in IgG, IgA, or IgM levels. Previous studies did not evaluate the effect of the sensitivity count on immunoglobulin levels. A study by Wahn et al. reported a correlation between the SCORAD index and the number of allergen sensitizations [24]. In our study, we also found that the SCORAD index was higher in patients with multiple sensitivities.

In a study by Dadkhah et al., the prevalence of AD was found to be 8.9% among 45 patients receiving IVIG treatment for hypogammaglobulinemia [25]. Yartsev et al. found that AD was the most common allergic disease in patients with IEIs [26]. In Wang’s study, IgG level normalization was observed in seven of eight patients within 2 years of hypogammaglobulinemia diagnosis [27].

In our investigation, hypogammaglobulinemia persisted in 86 (61%) of the 142 patients followed over a median period of 18 months (range 3–25 months). Importantly, IgG level normalization during follow-up was significantly more frequent in patients with isolated IgG deficiency than in those with concomitant low IgA and/or IgM levels (53.7% vs. 25.87%). This finding suggests that concomitant hypogammaglobulinemia may represent a more persistent or biologically distinct immunological condition requiring closer long-term monitoring. These findings support the clinical relevance of subclassifying patients according to accompanying immunoglobulin deficiencies. Notably, although baseline IgG levels were lower in the concomitant deficiency group, the persistence of hypogammaglobulinemia was more strongly associated with the presence of multiple affected immunoglobulin isotypes than with the absolute IgG level itself. The coexistence of low IgA and/or IgM levels may therefore reflect a deeper defect in humoral immunity and may indicate a lower likelihood of spontaneous normalization over time.

Griffin et al., in their recently published retrospective study, evaluated 24 AD patients with hypogammaglobulinemia alongside a cohort of 180 patients with AD; however, they did not demonstrate a significant association between AD severity and IgG levels [28]. This lack of association is not unexpected, given the very small subgroup sizes—particularly the presence of only eight patients with moderate AD and three with mild AD—which substantially limits the statistical reliability of comparisons across severity strata. In contrast, our study comprised a considerably larger cohort of 200 hypogammaglobulinemic AD patients, enabling more robust, adequately powered analyses. Notably, we observed that IgG, IgA, and IgM levels were significantly lower in patients with moderate-to-severe AD compared with those with mild disease.

Therefore, the principal strength of our study and its key contribution to the literature lies in demonstrating that hypogammaglobulinemia is associated not only with severe but also with moderate atopic dermatitis, suggesting a relationship between increasing AD severity and broader humoral immune alterations. These findings may broaden current perspectives on the association between AD severity and immunoglobulin levels, and underscore the need for increased clinical awareness and systematic immunological screening in this subgroup.

In a study by Yasuno et al., a significant association was found between immunoglobulin level normalization and AD symptom improvement in five patients with THI and AD. The skin findings of patients with AD resolved after their IgG levels returned to normal at 12 months of age, suggesting a relationship between THI and the occurrence of AD. Their study suggests that hypogammaglobulinemia in patients with AD may be attributed to a failure in production rather than protein loss from the skin and/or intestine [29]. Walker et al. reported that 12 of 15 THI patients had symptoms of atopic disease or food allergy/intolerance, and they suggested that subclinical intestinal protein loss due to allergic inflammation may contribute to the development of THI [30].

According to the Immune Deficiency Foundation, the estimated frequency of THI is approximately 1 in 1000 children; however, these figures are widely acknowledged to underestimate the true incidence, as immunoglobulins are not routinely measured in healthy children. Studies based on primary immunodeficiency registries indicate that THI accounts for approximately 2–2.3% of diagnosed IEI cases; however, these data do not reflect population-based prevalence [28,31]. In Kilic et al.’s two-center cohort, antibody deficiencies accounted for 73.5% of all IEIs, and THI accounted for 31% of primary antibody deficiencies (22.9% of all IEIs), suggesting a diagnosed THI prevalence of approximately 7 per 100,000 in Turkey [32]. This figure falls at the lower bound of Walker et al.’s international estimate (0.061–1.1 per 1000 live births), indicating that THI is likely substantially underdiagnosed in Turkey [30]. In the largest prospective European cohort (Italian Primary Immunodeficiency Network, IPINET), the IgG values of 72% of 57 children with an initial THI diagnosis spontaneously normalized within 24 months [33]. Similarly, Karaca et al. reported that 90.1% of 101 Turkish THI patients achieved IgG normalization at a mean of 29.2 ± 15.2 months [34]. Importantly, Sütçü et al., in an analysis of 91 THI patients focusing on predictive factors for recovery, found that IgG normalization occurred at a mean of 30.6 ± 11.88 months; 69.3% of patients recovered within the first 3 years of life, whereas 30.7% exhibited delayed recovery beyond this period. The presence of atopy, low initial IgA and IgM levels, and a history of more than six recurrent infections per year were significantly associated with late recovery [35]. In our cohort, only 56 of 142 patients (39%) achieved age-appropriate IgG normalization after a median follow-up of 18 months, a rate markedly lower than those reported in the aforementioned series. This discrepancy may be attributable to the shorter follow-up period, concurrent atopic dermatitis, and the young age structure of our population; however, it also underscores the need for prolonged immunological surveillance in this patient group. It should be noted that the 10.8% prevalence of hypogammaglobulinemia observed in our study reflects a selected atopic dermatitis population and cannot be directly compared with population-based THI incidence estimates.

One possible reason for the lack of a significant increase in infection frequency is that a large majority of patients (75%) had mild atopic dermatitis. Mild atopic dermatitis is associated with more limited epidermal barrier impairment, so a lower risk of secondary skin infections is expected. Furthermore, none of the patients showed clinical or immunological findings suggestive of serious combined immunodeficiency or other T-cell defects; this was supported by lymphocyte subgroup analyses that were normal in 92.5% of patients. In addition, the mean follow-up period of 18 months may have been insufficient to fully capture the spectrum of infection-related morbidity. The retrospective nature of the data collection may also have prevented the recording of infections treated outside our center. Our findings are consistent with those from previous studies on THI, which reported no significant increase in the frequency of serious infections in most children with THI. To more accurately and comprehensively assess the infection burden, prospective studies incorporating standardized infection surveillance are needed [36,37].

Although immunoglobulin therapy has been reported to improve AD symptoms in selected severe cases [38], immunoglobulin replacement therapy is not recommended for AD due to the lack of proven results and the concern that passive antibody administration may delay the maturation of humoral immunity [39,40,41]. In our study, none of the patients received immunoglobulin replacement therapy.

## 6. Conclusions

In our study, the prevalence of hypogammaglobulinemia among children with atopic dermatitis was significantly higher than the reported rates of THI in the general pediatric population. Moreover, the observed humoral immune abnormality was not limited to IgG deficiency alone. Patients with moderate-to-severe AD exhibited significantly lower levels of all three major immunoglobulin isotypes (IgG, IgA, and IgM) when compared with those with mild disease. Multivariable logistic regression analysis demonstrated that lower IgG levels were independently associated with moderate-to-severe AD after adjustments for age, sex, eosinophilia, total IgE levels, and food allergies. In addition, linear regression analysis identified food allergy as the strongest independent predictor of the SCORAD index. Our findings suggest that, in most patients, the observed immunological abnormalities may reflect a benign and potentially self-limited delay in immune maturation rather than a classical primary immunodeficiency. However, in patients with persistent hypogammaglobulinemia, early-onset severe AD, recurrent or unusual infections, or additional warning signs suggestive of an inborn error of immunity, further immunological and (when clinically indicated) genetic evaluation may be warranted.

Our study underscores the importance of systematic immunoglobulin screening, particularly in children with moderate-to-severe AD, and suggests that long-term immunological follow-up may be beneficial in patients with persistent immunoglobulin deficiency. We also believe that more multicenter, multi-ethnic prospective studies with standardized follow-up protocols are needed to better characterize the long-term immunological and clinical outcomes in children diagnosed with atopic dermatitis.

## Figures and Tables

**Table 1:** Distribution of immunoglobulin values by age group.

Patient Age Group and Number	IgG (mg/dL) Median (IQR 25–75%)	IgA (mg/dL) Median (IQR 25–75%)	IgM (mg/dL) Median (IQR 25–75%)	Total IgE (IU/mL) Median (IQR 25–75%)
3–6 months (*n*: 56)	332 (300–429)	11.5 (10–18)	42 (29–62)	15 (5–49)
7–12 months (*n*: 81)	336 (300–395)	12 (10–16)	43 (30–56)	29 (12–87)
13–48 months (*n*: 55)	496 (353–561)	25 (16–35)	65 (57–63)	69 (7–96)
>49 months (*n*:8)	585 (480–667)	57 (34–92)	110 (80–143)	60 (40–182)

**Table 2:** The effects of food allergies, allergen sensitization diversity, and clinical follow-up duration on immunoglobulin distribution.

Immunoglobulins	Food Allergy			Allergen Sensitivity			Immunoglobulin Change		
	Yes Median (IQR 25–75%)	No Median (IQR 25–75%)	*p*	Monosensitization Median (IQR 25–75%)	>2 Allergens Sensitization Median (IQR 25–75%)	*p*	First Visit Median (IQR 25–75%)	Last Visit Median (IQR 25–75%)	*p*
Total IgE (IU/mL)	54 (16.5–162)	15 (5.6–38.2)	<0.001	16 (6–48)	38 (15–137)	0.002	38 (10–149)	115 (20–180)	0.131
IgG (mg/dL)	327 (300–427)	367 (300–477)	0.213	327 (300–492)	334 (300–413)	0.264	324 (300–396)	521 (367–647)	<0.001
IgA (mg/dL)	13 (10–19)	12 (10–24)	0.881	14 (10–18)	12 (10–19)	0.283	11 (10–16)	22 (13–35)	<0.001
IgM (mg/dL)	43 (27–61)	46 (33–66)	0.192	43 (29–68)	44 (30–59)	0.214	41 (29–57)	64 (46–88)	<0.001

**Table 3:** Factors affecting the SCORAD index.

	SCORAD Index			
		Median (IQR 25–75%)	*p*	
Gender	Male (*n* = 128)	21.0 (17–25)	0.294	
	Female (*n* = 72)	19.0 (16–36)		
Food allergy	Yes (*n* = 72)	23.0 (20–57)	<0.001	
	No (*n* = 128)	18.5(16–22)		
	SCORAD			
	Mild	Moderate–severe	*p*	Effect size (r)
Eosinophil count (10^3^/µL) median (IQR 25–75%)	280 (170–470)	410 (317–587)	<0.001	0.332
Total IgE IU/mL median (IQR 25–75%)	21 (6–66)	26 (12–73)	0.462	0.052
IgG (mg/dL) median (IQR 25–75%)	374 (300–488)	300 (300–363)	<0.001	−0.346
IgA (mg/dL) median (IQR 25–75%)	14 (10–25)	10 (10–16)	0.006	−0.268
IgM (mg/dL) median (IQR 25–75%)	51 (36–70)	38 (26–57)	0.001	−0.319
Vitamin B12 (ng/L) median (IQR 25–75%), normal range (197–771)	350 (158–461)	222 (104–340)	0.042	0.243
25-hydroxy vitamin D (µg/L) median (IQR 25–75%), normal range (30–80)	33.7 (26–44)	28.0 (22–37)	0.981	0.143

Effect size reported as rank-biserial correlation (r). Interpretation: *r* = 0.1 small, *r* = 0.3 medium, *r* = 0.5 large.

**Table 4:** Multivariate logistic regression analysis of factors associated with moderate-to-severe atopic dermatitis.

Variable	OR	95% CI	*p*	VIF
Age (months)	1.029	0.999–1.060	0.062	1.74
Sex (female)	0.717	0.314–1.638	0.430	1.15
Eosinophil count *	1.424	0.877–2.311	0.153	1.18
Total IgE *	1.059	0.814–1.378	0.668	1.34
Food allergy	1.923	0.882–4.192	0.100	1.26
IgG (mg/dL)	0.993	0.988–0.999	0.013	1.79
IgA (mg/dL)	1.000	0.984–1.016	0.994	2.75
IgM (mg/dL)	0.985	0.968–1.003	0.111	2.97

* Log-transformed values; OR, odds ratio; CI, confidence interval; VIF, variance inflation factor.

**Table 5:** Multiple linear regression analysis of factors associated with the SCORAD index.

Variable	B	95% CI	*p*
Age (months)	0.096	−0.082–0.274	0.288
Sex (female)	−1.386	−6.734–3.961	0.610
Eosinophil count *	2.430	−0.802–5.662	0.140
Total IgE *	−0.023	−1.831–1.786	0.980
Food allergy	11.972	6.408–17.536	<0.001
IgG (mg/dL)	−0.029	−0.058–0.001	0.055
IgA (mg/dL)	0.022	−0.038–0.081	0.472
IgM (mg/dL)	−0.055	−0.125–0.015	0.124

* Log-transformed values; B, unstandardized regression coefficient; CI, confidence interval.

**Table 6:** Comparison of patients with isolated IgG deficiency and concomitant low IgA and/or IgM levels.

Variable	Isolated Low IgG (n = 61)	Concomitant Low IgA and/or IgM Levels (n = 139)	*p*
Gender Male, *n* (%) Female, *n* (%)	37 (60.7%) 24 (39.3%)	91 (65.5%) 48 (34.5%)	0.67
Age, months, median (IQR 25–75%)	8 (6–19)	8 (5–14.5)	0.316
Mild AD, *n* (%)	50 (82.0%)	100 (71.9%)	0.184
Moderate–severe AD, *n* (%)	11 (18.0%)	39 (28.1%)	0.184
Food allergy, *n* (%)	19 (31.1%)	55 (39.6%)	0.329
SCORAD, median (IQR 25–75%)	19 (17–24)	20 (15.5–37)	0.915
IgG mg/dL, median (IQR 25–75%)	399 (300–496)	336 (300–425)	0.014
Total IgE IU/mL, median	25.5	22.5	0.461
Eosinophils/μL, median	255	350	0.066
IgG recovery, *n* (%)	22/41 (53.7%)	24/101 (25.8%)	0.001

## Data Availability

The data that support the study findings are available from the corresponding author upon reasonable request.

## Abbreviations

AD	Atopic dermatitis.
IQR	Interquartile range.
Ig	Immunoglobulin.
SCORAD	Scoring Atopic Dermatitis.
IVIG	Intravenous immunoglobulin.
STAT	Signal transducer and activator of transcription.
IEI	Inborn error of immunity.
THI	Transient hypogammaglobulinemia of infancy.
IPEX	Immune dysregulation, polyendocrinopathy, enteropathy, X-linked.
GOF	Gain-of-function.
SD	Standard deviation.
